# Psychosocial Work Environment and Teachers’ Psychological Well-Being: The Moderating Role of Job Control and Social Support

**DOI:** 10.3390/ijerph18147308

**Published:** 2021-07-08

**Authors:** R Zirwatul Aida R Ibrahim, Wan Zaleha Mohd Zalam, Bob Foster, Teuku Afrizal, Muhamad Deni Johansyah, Jumadil Saputra, Azlina Abu Bakar, Mazidah Mohd Dagang, Siti Nazilah Mat Ali

**Affiliations:** 1 Faculty of Business, Economics and Social Development, Universiti Malaysia Terengganu, Kuala Nerus 21030, Terengganu, Malaysia; zirwatul@umt.edu.my (R.Z.A.R.I.); wanzaleha_91@yahoo.com (W.Z.M.Z.); mazidah@umt.edu.my (M.M.D.); nazilah@umt.edu.my (S.N.M.A.); 2 Faculty of Economics and Business, Universitas Informatika dan Bisnis Indonesia, Bandung 40285, West Java, Indonesia; bobriset@unibi.ac.id; 3 Faculty of Social and Political Sciences, Universitas Diponegoro, Semarang 50275, Jawa Tengah, Indonesia; 4 Faculty of Mathematics and Natural Sciences, Universitas Padjadjaran, Jatinangor, Sumedang 45363, West Java, Indonesia; muhamad.deni@unpad.ac.id; 5 Faculty of Human Development, Universiti Perguruan Sultan Idris, Tanjong Malim 35900, Perak, Malaysia; azlina.ab@fpm.upsi.edu.my

**Keywords:** psychosocial work environment, psychological well-being, teachers, job control, social support, moderated analysis

## Abstract

Nowadays, the issue of teachers’ psychological well-being causes serious concern, especially in Malaysia. Many studies related to psychological well-being have focused on students rather than on the health and well-being of teachers. Thus, the current study investigated the determinants of psychological well-being (depression, anxiety and stress) from the psychosocial work environment (job control, job demands and social support), and examined the moderating role of job control and social support in the relationship between job demands and psychological well-being among teachers. The design of this study was quantitative research through a survey questionnaire. The sample consisted of 335 high school teachers (23.3%—male; 76.7%—female) who responded to measuring scales of job demands, job control, social support, depression, anxiety and stress, and socio-demographic profile. The data were analyzed using two statistical methods, namely descriptive and inferential statistics. The hierarchical linear regression model was used to analyze the data by assisting the statistical software, i.e., SPSS-23. The results showed that job demands, job control and social support significantly predicted teachers’ psychological well-being. Furthermore, the effect of job demands on teachers’ depression and anxiety was partially moderated by job control and social support. In conclusion, this study has successfully identified the significant predictors of teachers’ psychological well-being and the role of job control and social support as a moderating variable to teachers’ psychological well-being in Malaysia. The result provides insights and contributes to the literature of teachers’ psychological well-being determinants and involves Malaysian respondents with a collectivistic eastern culture.

## 1. Introduction

Education is crucial to achieving the third goal of the Sustainable Development Goals (SDGs) related to good health and well-being. As emphasized by the United Nations Educational, Scientific and Cultural Organization [1], education is key to educating people on drug and alcohol abuse and prevention, and mental health issues. In addition, it provides relevant knowledge and information on family planning, sex education and reproductive health. Teachers are in charge of preparing the coming generations to face the challenges of current unsustainable development [2]. Therefore, teachers need to preserve and maintain their psychological well-being before dealing with the various challenges in the education sector. Due to this awareness, the Ministry of Education, Malaysia, has taken the initiative by providing exposure to techniques and skills in emotional management that can improve psychological well-being among teachers through the “Raising Teachers Psychological Well-being Awareness #3A (Aware, Alert, Action) Program”.

Many international studies over the last decades have highlighted the stressful nature of teaching [3,4,5,6]. There is a current need to prioritize teachers’ occupational mental health problems, especially in Malaysia. Workers’ mental health status has been shown to vary depending on their job [7]. Apart from teaching duties, teachers nowadays are burdened with a heavy load of administrative duties daily, such as documentation and conducting programs [8,9]. Teaching, in particular, has longer hours, a lot of work, and is emotionally demanding for the government, students, and parents [7]. A normal teacher’s day begins early in the morning and consists of roughly five lessons, including extracurricular activities and other teaching responsibilities, such as assessments and reports. Because many teachers are graded on the objective results that marks can provide for evaluation purposes, marking exercise books consumes a significant amount of nonteaching time [10]. Teachers have begun to express concern about their ability to control their students. Although teachers will inevitably experience a high level of stress while working, the stress in the workplace can have a long-term impact on their health and well-being [11,12]. Attending to the physical well-being of teachers could contribute to their feelings of detachment, absenteeism, and the desire to leave their occupation [13].

Research data by Musa, Moy and Wong [8] have proven that most Kuala Lumpur and Selangor teachers need to enter student data and examination scores into the online system after work hours (7:30 a.m. to 1:00 p.m.) as the system may experience congestion during working hours. Sometimes teachers may have to wait until midnight for the online system to be less congested, reducing their sleep time. It would eventually affect the learning environment of students and hinder the attainment of educational objectives by governments. While protecting the well-being of teachers has an impact beyond the teaching profession, it is also critical to the general public. Social epidemiological explanations for the health effects of the work environment suggest that certain job characteristics increase the worker’s susceptibility to job strain, which has negative consequences for mental and physical health [14].

A report from the National Institutes of Health, Ministry of Health Malaysia [15] has revealed that as many as 2.3 percent of adults in Malaysia suffer from depression. With regards to the teaching profession, depression among teachers in Malaysia is very serious as a result of the increasing workload [16]. Surprisingly, the National Union of the Teaching Profession also revealed that diligent teachers are the ones who suffer from high levels of work stress due to the overwhelming workload, based on their great effort [17,18]. In 2018, the Malaysian Education Minister, Maszlee Malik, reported that 4.4 percent of the 48,258 teachers were identified as experiencing moderate stress levels due to family and financial matters, management, workload, not being appreciated, lack of skills in carrying out tasks, as well as not being trained to address stress issues [19,20,21]. In line with previous studies, teaching has been proven to be a demanding and stressful profession due to the physical and mental challenges [22,23,24].

## 2. Literature Review

### 2.1. Teacher’s Psychological Well-Being

Psychological well-being or mental health among teachers has become an increasing issue in many countries [25,26,27]. Nevertheless, most studies in school settings have been on students rather than on the health and well-being of teachers [28]. A recent systematic review also suggested that studies among school teachers in Malaysia of the occupational risk factors (such as teaching experience, ergonomic issues, workload, and salary) of illness and stress are still lacking [29]. Based on previous studies, several factors contribute to stress, anxiety and depression among teachers, such as age, higher teaching experience, inadequate salaries, higher qualifications, higher workload and psychological job demands [24,30]. A study of secondary school teachers in Nigeria by Iyore [31] confirmed that work overload, crowded class conditions, poor working conditions, lack of social support and lack of teaching accessories are sources of stress. Besides that, income and salary were considered common risk factors for stress, with teachers with a higher salary or income experiencing a lower prevalence of stress compared with those with a lower salary [29]. Contrary findings were recorded in India, where teachers with higher salaries were found to suffer from high levels of stress [32].

In Malaysia, a study conducted by Othman and Sivasubramaniam [33] found that teachers had a high prevalence of depressive (43.0%), anxiety (68.0%) and stress (32.3%) symptoms. While teachers had severe to extremely severe depression, anxiety and stress were reported by 9.9 percent, 23.3 percent and 7.0 percent. Teachers with socio-demographic and work-related characteristics such as being female, having a low educational status, having 1–3 children, staying with in-laws, shorter distance to school, living in a high-rise building and owning a house are more likely to experience depression, anxiety and stress.

Furthermore, Samad, Hashim, Moin and Abdullah [34] reported that most of the primary school teachers in Klang, Malaysia, experiences moderate stress levels (71.7%) due to student misbehavior. Female teachers with heavy workloads in school or home were most likely to have poor mental health. Meanwhile, Ghani, Ahmad and Ibrahim [25] concluded that special education teachers suffered mild stress and indicated student misbehavior as the primary source of teacher stress, followed by workload, time and resources difficulties, recognition, and interpersonal relationships. A comparative study by Kavita and Hassan [23] on teachers in Selangor revealed that secondary school teachers, compared with primary school teachers, perceived more stress in all stress factors, which were rapport with parents and co-workers, workload, time constraints, student attitude, recognition and support, and lack of resources. Teachers with teaching experience between 11–15 years experienced more stress and teachers aged between 31–50 years experienced more stress than younger teachers aged 20–30 years and older teachers aged 51–60 years. Thus, the researcher suggested investigating the most influential motivational factors (intrinsic and extrinsic) that lead to teachers’ superior performance.

Generally, high levels of stress can create physical, psychological and behavioral problems among teachers, such as absenteeism, early retirement, sickness, tardiness, depression, insomnia, and attrition. Then, there is the impact on family life [35,36]. Velaytham and Surat [22] indicated that high occupational stress would result in higher work−family conflict among teachers, where teachers have to change plans with family members because of their job requirements. Iyore [31] concluded that stress increases attrition, reduces the quality of instructional delivery, lowers morale and job satisfaction, as well as job performance among teachers.

A qualitative study by Shernoff, Mehta, Atkins, Torf, and Spencer [37] found that stress manifests in urban teachers and significantly impacts their sense of efficacy, job satisfaction, burnout, attrition, student engagement and physical health. In terms of physical health, teachers reported frequent illness, sleep difficulties, unhealthy eating habits and frequent exhaustion. Furthermore, Musa, Moy and Wong [8] highlighted that stress is a significant factor in sleep quality. The study was conducted on public secondary schools from Kuala Lumpur and Selangor and showed that teachers with stress had poor sleep quality. Teachers were also reported to suffer from depression and anxiety, affecting their sleep quality, due to overthinking about their students’ academic performance, which forms part of their key performance index. According to Moy, Hoe, Hairi, Buckley, Wark, Koh and Bulgiba [28], stress linked with major chronic medical conditions such as obesity, hypertension, impaired glucose tolerance, diabetes mellitus, coronary heart diseases, kidney failure and cancers among teachers. Apart from stress, teachers in Malaysia who reported depression and anxiety were more likely to experience musculoskeletal pain (low back pain and neck and/or shoulder pain) [38,39].

### 2.2. Job Demand and Psychological Well-Being

Job demands are defined as the psychological stressors involved in accomplishing the workload, stressors related to unexpected tasks, and stressors of job-related personal conflict [40]. Generally, previous studies have illustrated that high demands and workloads are a significant predictor of mental health which has positive impact on depression and stress [41,42] as well as burnout among employees [43,44,45,46]. Kinman, Clements and Hart [47] reported that job demands were strong predictors of mental health problems such as anxiety/insomnia, depression, social dysfunction and somatic symptoms among prison officers in the United Kingdom. In studies of job demand, interpersonal conflicts at work, organizational constraints and workload have emerged as relevant stressors and strains to mental and physical health [48,49,50]. Workload is listed as one of the most common sources of stress [51]. Numerous studies have pointed out the correlation between a demanding workload and poor physical and mental well-being [45,49].

In terms of job demands in the context of teacher work, these groups are at high risk of experiencing health problems such as depression, anxiety and stress [52,53]. Case in point, a study by Ibrahim, Zalam, Dagang, Omar, Bakar and Ali [54] of teachers in the East Coast, Malaysia, reportedly faced job demands that included a large number of students who needed to be taught, as well as teaching tasks and ever-changing administrative work that affected their mental health. They also faced a high risk of stress to maintain high-profile school performance. Van Droogenbroeck, Spruyt and Vanroelen [55] reported that teaching-related and non-teaching-related workload consequently elicited negative responses, including burnout among senior teachers in Belgium. In a similar study done by Baka [56], a study of 316 Polish teachers applying the Job Demands-Resources model reported that job demands (interpersonal conflicts at work, organizational constraints, quantitative workload) had a stronger effect on mental health than on physical health.

Similar findings were also recorded in other sectors. For example, studies involving nurses in Japan and Korea report that nurses are at high risk of depression due to specific work stress rates in the nursing sector [57,58]. Other studies also revealed that job demands, including verbal assault by citizens, workload, and administrative stressors, have caused police officers in Germany to experience emotional exhaustion [48].

### 2.3. Job Control and Psychological Well-Being

Job control, also referred to as decision latitude, is defined as a working individual’s potential control over his tasks and conduct during the working day [40,59]. Two separate studies involving prison officers in the United Kingdom and Iran reveal that low job control was a critical predictor of mental health status [47,60]. In a similar manner, Yakub and Sidik [61] found that when job control was decreased, the course led to work stress and anxiety among employees. Apart from that, psychosocial factors, including job control and social support, have negatively affected mental (depression and emotional exhaustion) and physical health (musculoskeletal disorders) among teachers in Malaysia [38,62]. Recently, Ibrahim et al. [54] pointed out that lack of job control led to teachers’ psychological distress in Malaysia’s eastern coast.

### 2.4. Social Support and Psychological Well-Being

Social support can be considered a product of the social dimension of lifestyle, especially related to mental health [63]. In the workplace environment, depression among employees may be minimized by more social support because they can defend themselves against the adverse effects of life [64]. Case in point, employment management and supervisors support are two main factors that reduce nurses’ depression in Japan [65]. In the context of the education sector in Malaysia, teachers with low social support will be more likely to have high psychological distress [54]. As a social unit that can help to improve mental health, the maximum focus is therefore critical in providing teachers with social support. This is due to teachers needing positive social support, encouragement and effective communication from school leaders and colleague teachers to enhance their well-being in the workplace [54,66].

### 2.5. Social Support as a Moderator

Brough, O’Driscoll and Biggs [67] asserted that social support serves as a moderator in stress relationships, where individuals with strong support can avoid the negative effects of stress. In the workplace, social support can provide the prevention of psychosocial stress and enhance social relationships. Blanch [68] explains that social support can provide a consistent role as a moderator in the relationship between job control and job stress. Social support is defined as “the resources provided by other persons”, including family members, friends, supervisors and colleagues [69,70]. Generally, social support is a process of interaction in increasing self-esteem, sense of ability and good coping strategies, and being able to demonstrate real competence or accept change from physical or psychosocial sources [71].

Due to the limited and inconsistent findings of the role of social support as a buffer in the job demands-control-support (JDCS) hypothesis, further studies need to be conducted [72]. Chay [73] performed a study involving 117 entrepreneurs and confirmed that social support in the workplace has a strong buffering effect that can reduce stress and improve physical and psychological well-being. In the study, employees with high social support were slightly affected by low work discretion, while those with low support were more likely to suffer from psychological illness. Similarly, Chen, Siu, Lu, Cooper and Phillips [74] found that social support acts as a moderator in the relationship between job stress and depression.

Roxana [75] also proves that job satisfaction increases, and stress can be reduced when employees receive support from colleagues and supervisors. Grandey [76] argues that employees who receive high support from colleagues and supervisors are less affected by adverse emotional effects. Grandey [76] also stressed that support from supervisors could reduce negative emotions by creating a positive work environment, where it is easier for employees to feel and express positive emotions that the organization does not expect. Thus, researchers can conclude that social support is an essential source as a moderator role for employee health and organizational well-being. Most of the previous studies focused on organizational goals, with limited research on aspects of employee health, namely social support as a moderation to psychological well-being. Therefore, this study should be conducted to study the role of social support as a moderator in the relationship of the psychosocial work environment and psychological well-being.

In conjunction with the previous elaboration, among the widely and the most influential theoretical frameworks that relate the characteristics of a job to health and wellbeing is the job demand-control model [38]. Thus, the current study adopts the JDC and JDCS models by testing the buffer hypothesis testing, i.e., thee job resources’ moderating role (job control and social support) on the relationship between job demands on teachers’ psychological well-being. Furthermore, since the current study focuses on the main, additive and buffering effect (moderating effect hypothesis) instead of the mediator effect hypothesis, the JDC and JDCS models are the most appropriate theoretical background for this study instead of JDR.

## 3. Materials and Methods

### 3.1. Research Design

This study is designed using a quantitative approach, where in any circumstance the practical insights are vital and statistical findings are required. Quantitative research provides a better way to identify the issues and problems in an organization [77]. For a company to grow, quantitative research methodologies are important. When making decisions relating to the future of the organization, insights obtained from hard numerical data and analysis are quite helpful. There are two types of quantitative research utilized in this study, namely descriptive and explanatory design. In a descriptive design, a researcher is only interested in explaining the scenario or case they are studying [78]. This design was built by analyzing, compiling, and presenting obtained data. Descriptive design helps people to comprehend the need for research. If the problem statement is unclear, perform an exploratory investigation. The exploratory investigation is designed on the basis of a researcher’s views and thoughts about a subject that are used to further examine their hypotheses through the usage of explanatory design [79]. The study helps to uncover facets of a subject that have not yet been addressed. The quantitative approach applies a cross-sectional study. It is a sort of observational research, where the data on variables are analyzed at a single point in time across a sample population or predefined subgroup [80].

### 3.2. Participants

The research included 78 (22.3%) male and 257 (76.7%) female high school teachers in Kuala Terengganu, a district in the state of Terengganu, Malaysia. The ages ranged from 31 and 50 years, with an average of 40.5. The majority of participants were married (91.6%), followed by single or divorced (8.4%); 89.6% of participants had a least one child or more, and 10.4% had no child. A large number of participants were Malay Muslim (96.4%), and others were Chinese (2.7%) and Indian (0.9%). With regards to working experience, 268 (80%) teachers had been working for 11 years or more, 57 (17%) for 6 to 10 years and 10 (3%) for 3 to 5 years (see Table 1).

### 3.3. Research Instruments

All measures were back-to-back translated from English to Malay version by independent translators and experts [81]. The questionnaires were divided into three parts. The first part included participants’ demographic profile, including sex, age, ethnicity, marital status, monthly income and level of education. The second and third part included the instruments measuring job demands, job control and social support (see Appendix A), and psychological well-being (see Appendix B).

#### 3.3.1. Job Demands, Job Control and Social Support

Twenty-two items of the Job Content Questionnaire (JCQ) were used to assess job demands, job control and social support [82]. The items were rated on a 4-point Likert scale ranging from 1 = strongly disagree to 4 = strongly agree, with a higher score indicating higher demands, control and support. The Cronbach’s alpha for the current study were good: 0.79 for job control and 0.84 for social support, and minimum acceptable (Cronbach’s alpha is 0.65) for psychological demands [83,84].

#### 3.3.2. Psychological Well-Being

Psychological well-being was measured using the Depression, Anxiety and Stress Scale (DASS) developed by Lovibond and Lovibond [85]. Twenty-one items with three distinctively domains achieved good internal consistencies: 0.86 for depression, 0.88 for anxiety and 0.86 for stress. These items ranged from 0 = did not apply to me at all 3 = applied to me very much or most of the time. The summary of the DASS score is presented in Table 2.

### 3.4. Data Analysis

The survey data were collected using questionnaire with paper and pencil and during working hours from high school teachers in Kuala Terengganu, a district in the state of Terengganu, Malaysia and analyzed by using two statistical techniques, i.e., descriptive and inferential statistics. The descriptive statistics consists of frequency, percentage, mean and standard deviation. For inferential statistics, this study uses the hierarchical linear regression model (HLRM). HLRM was employed to test the main, additive and moderating effect of the proposed hypotheses [86]. Demographic variables, namely gender, age, number of children, marital status and work experience were entered into the model as control variables. Subsequently, the HLRM includes job demands, job control and social support to test the additive effect as well as the moderating effect (job demands × job control and job demands × social support) in describing variance in the psychological well-being of teachers. In addition, the data analysis was performed by utilizing the statistical software, i.e., statistical package for the social sciences SPSS version 23 (IBM Inc., New York, NY, USA)

## 4. Results

### 4.1. Descriptive Statistics

Before embarking on inferential tests, such as correlation and hierarchical regression analyses, this study reports and elaborates descriptive statistics that involved mean and standard deviation. This study will also conclude the actual condition and level of studied variables. The results of descriptive statistics can be seen in Table 3.

Table 3 displays the results of descriptive statistics for the studied variables. The results show the level of depression categorized as extremely severe with a mean value of 28.06, and the standard deviation equal to 2.47. Anxiety mean value was 27.47, with a standard deviation of 2.72. Thus, it can be concluded that the level of anxiety was categorized as extremely severe. For stress, the mean value was 23.39 with a standard deviation of 2.40 and also classified as extremely severe. In addition, this study also reported the severity level of psychological well-being (depression, anxiety and stress). 

Table 4 captures the results of the severity levels of psychological well-being (depression, anxiety and stress). The severity levels of the teachers’ psychological well-being were that a total of 43.59% (*n* = 146) teachers reported having severe depressive symptoms while the percentage having extremely severe symptoms was 25.69% (*n* = 86). Roughly 0.57% (*n* = 2) and 30.15% (*n* = 101), respectively, had mild or moderate symptoms. Some 82.39% (*n* = 276) were classified as extremely severe and 14.93% (*n* = 50) were in the severe class, while the remaining were at mild (0.89%; *n* = 3) and moderate (1.79%; *n* = 6) levels in terms of anxiety symptoms. Some 93.43% (*n* = 313) of teachers felt stress, with 53.13% of these (*n* = 178) in the mild to moderate category and 40.3% (*n* = 135) being in the severe to extremely severe category.

Table 5 displays the results of the descriptive statistics and level of job control, job demands and social support. The results indicated the level of job control categorized as medium with a mean value was 3.26, and the standard deviation was equal to 0.30. The job demands mean value was 2.72, with a standard deviation of 0.32. Thus, it could be concluded that the level of job demands was categorized as medium. For social support, the mean value was 3.07 with a standard deviation of 0.22 and classified as medium.

### 4.2. Correlation of Studied Variables

Internal consistencies and zero-order correlations of the studied variables were presented in Table 6. As expected, most variables were significant and correlated to each other.

Table 6 displays the results of the correlation matrix for all studied variables. This study used three dimensions for measuring psychological well-being, namely depression, anxiety and stress (DASS) and assigned as dependent variable. Then, three other constructs were job demands, job control and social support. This study uses Cronbach Alpha (CA) in assessing the reliability of measurement scales. Hair et al., 2014 stated that the constructs are reliable when the value of Cronbach Alpha is higher and equal to 0.60. Of these, the results of the analysis showed that the minimum value of CA was 0.65 (job demands) and maximum value was 0.88 (anxiety). It means that all studied variables or constructs are reliable.

Furthermore, the results of the correlation matrix showed that job demands had a positive and significant correlation with psychological well-being, i.e., depression (*r* = 0.22), anxiety (*r* = 0.20) and stress (*r* = 0.21) at the level of *p* < 0.01. Job control had a negative and significant correlation with psychological well-being, i.e., depression (*r* = −0.21), anxiety (*r* = −0.23) and stress (*r* = −0.24) at the level of *p* < 0.01. Social support had a negative and significant correlation with psychological well-being, i.e., depression (*r* = −0.07), anxiety (*r* = −0.07) and stress (*r* = −0.07) at the level of *p* < 0.05.

### 4.3. Additive and Moderating Effects

The analysis of hierarchical linear regression model used to predict the additive effects of job demands, job control and social support on psychological well-being, and the moderating effects of job control and social support in the relationship between job demands and psychological well-being can be seen in Table 7, Table 8 and Table 9.

#### 4.3.1. Social Support as a Moderator in the Relationship between Job Demands and Job Control with Depression

In this section, this study reports the results of the hierarchical linear regression model in predicting the teachers’ depression (see Table 7). There were three steps of analysis and involved control variables, such as gender, age, marital status, no. of children, and work experience. Then, predictor variables consisting of job demands, job control and social support, and two-way interaction, including job demands x social support and job control x social support

Table 7 shows the results of the hierarchical linear regression model in predicting depression. The control variables (gender, age, marital status, number of children and work experience) tested in the first step did not significantly contribute to depression (R^2^ = 0.019, F (5,329) = 1.241, *p* > 0.05). In the second step, job demands, job control and social support were included in the equation model, which described 10.4% of the total variance that contributed significantly to depression (F (8, 326) = 4.718, *p* < 0.001). The value of the standardized regression coefficient of job demands was 0.171 and significant at the level *p* < 0.01. It means that by assuming an increase in job demands of 1 percent would give an effect on increasing depression by as much as 17.1 percent. Further, job control was −0.197 and significant at the level *p* < 0.001. It means that by assuming an increase in job control of 1 percent would give an effect on reducing depression by as much as 19.7 percent. Social support was −0.110 and significant at the level *p* < 0.05. It means that by assuming an increase in social support by 1 percent, it would give an effect of reducing depression by as much as 11 percent. This suggests that job demands, job control and social support significantly predict depression. In the third step, social support moderated the relationship between job control and depression (β = 0.117, *p* < 0.05). This interaction led to a significant improvement in this regression model (∆R^2^ = 0.018, F (10, 324) = 4.509, *p* < 0.05).

The two-way interaction model for job demands and social support was not significant for depression (β = 0.064, *p* > 0.05). The value of the standardized regression coefficient of job demands was 0.163 and significant at the level *p* < 0.01. It means that by assuming an increase in job demands of 1 percent, it would give an effect of increasing depression by as much as 16.3 percent. Further, job control was −0.209 and significant at the level *p* < 0.001. It means that by assuming an increase in job control of 1 percent, it would give an effect on reducing depression as much as 20.9 percent. Social support was −0.132 and significant at the level *p* < 0.05. It means that by assuming an increase in social support of 1 percent, it would give an effect on reducing depression by as much as 13.2 percent.

**Figure 1:**
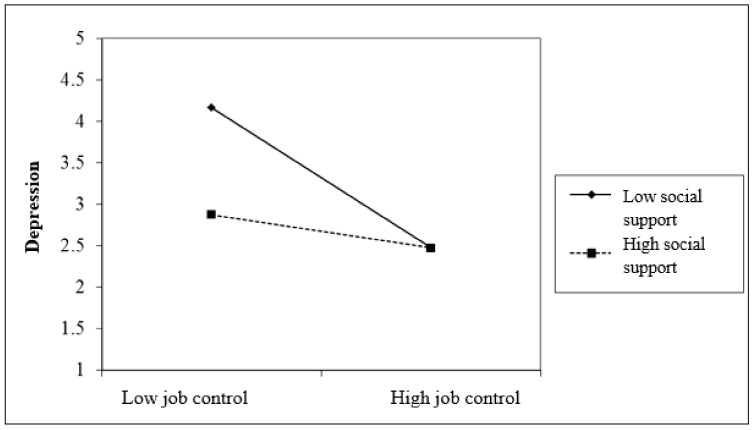
Two-way interaction of job control and social support in predicting depression.

Figure 1 illustrates the visual inspection of the interaction plot for the relationship between job control with depression and social support as a moderator (low, high). The results of regression analysis showed that job control had a more significant relationship to depression in the group of respondents with lower social support (*r* = 0.387, *p* < 0.001; β = 0.272) compared to the high social support group (*r* = 0.246, *p* < 0.01; β = 0.140).

#### 4.3.2. Social Support as a Moderator in the Relationship between Job Demands and Job Control with Anxiety

Table 8 shows the results of the hierarchical linear regression model in predicting anxiety. In the first step of the analysis, the control variables did not contribute significantly to anxiety (R^2^ = 0.026, F (5, 329) = 1.760, *p* > 0.05). Adding job demands, job control, and social support in step two contributed 11.1% variance in explaining anxiety (F (8, 326) = 5100, *p* <0.001). The value of the standardized regression coefficient of job demands was 0.143 and significant at the level *p* < 0.001. It means that by assuming an increase in job demands by 1 percent, it would give an effect on increasing the anxiety as much as 14.3 percent. Further, job control is −0.222 and significant at the level *p* < 0.01. It means that by assuming an increase in job control by 1 percent, it would give an effect on reducing anxiety by as much as 22.2 per-cent and social support is −0.109 and significant at the level *p* < 0.05. It means that by assuming an increase in social support of 1 percent, it would give an effect on reducing anxiety as much as 10.9 percent.

In the third step, social support moderated the relationship between job demands and anxiety (β = 0.110, *p* < 0.05). This interaction led to a significant improvement in this regression model (∆R^2^ = 0.017, F (10, 324) = 4.753, *p* <0.05). Meanwhile, the two-way interaction model for job control and social support was not significant for anxiety (β = 0.061, *p*> 0.05). The value of the standardized regression coefficient of job demands was 0.134 and significant at the level *p* < 0.001. It means that by assuming an increase in job demands of 1 percent, it would give an effect on increasing anxiety by as much as 13.4 percent. Further, job control is −0.228 and significant at the level *p* < 0.01. It means that by assuming an increase in job control of 1 percent, it would give an effect on reducing anxiety by as much as 22.8 percent; and social support is −0.113 and significant at the level *p* < 0.05. It means that by assuming an increase in social support 1 percent, it would give an effect on reducing anxiety by as much as 11.3 percent.

**Figure 2:**
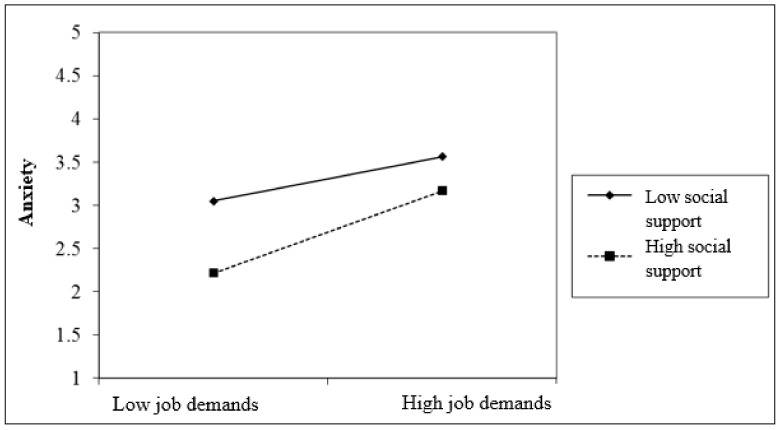
Two-way interaction of job demands and social support in predicting anxiety.

Figure 2 showed that job demands had a more significant relationship to anxiety in the group of respondents with lower social support (*r* = −0.381, *p* < 0.001; β = −0.157) compared to the high social support group (*r* = −0.250, *p* < 0.01; β = −0.120).

#### 4.3.3. Social Support as a Moderator in the Relationship between Job Demands and Job Control with Stress

Table 9 displays the results of the hierarchical linear regression model in predicting stress. There were three steps of analysis and involved control variables such as gender, age, marital status, no. of children, and work experience. Then, the predictor variables consisted of job demands, job control and social support. The two-way interaction included job demands x social support and job control x social support.

Table 9 shows the results of the hierarchical linear regression model in predicting stress. This study found that the control did not contribute significantly to the stress (R^2^ = 0.011, F (5, 329) = 0.707, *p* > 0.05). The results of the analysis showed job demands, job control, and social support contributed significantly (∆R^2^ = 0.098, F (8, 326) = 4.985, *p* < 0.001) to teachers’ stress. The value of the standardized regression coefficient of job demands was 0.157 and significant at the level *p* < 0.01. It means that by assuming an increase in job demands of 1 percent, it would give an effect on increasing stress by as much as 15.7 percent. Further, job control was −0.234 and significant at the level *p* < 0.001. It means that by assuming an increase in job control of 1 percent, it would give an effect on reducing stress by as much as 23.4 percent; and social support was −0.122 and significant at the level *p* < 0.05. It means that by assuming an increase in social support of 1 percent, it would give an effect on reducing stress by as much as 12.2 percent. However, social support did not moderate (F (10,324) = 4.348, *p* > 0.05) the relationship between job demands and stress as well as job control and stress relationships. The value of the standardized regression coefficient of job demands was 0.152 and significant at the level *p* < 0.01. It means that by assuming an increase in job demands of 1 percent, it would give an effect on increasing stress by as much as 15.2 percent. Further, job control was −0.244 and significant at the level *p* < 0.001. It means that by assuming an increase in job control of 1 percent, it would give an effect on reducing stress by as much as 24.4 percent; and social support is −0.141 and significant at the level *p* < 0.05. It means that by assuming an increase in social support of 1 percent, it would give an effect on reducing stress by as much as 14.1 percent.

## 5. Discussion

Our results revealed that job demands, job control and social support significantly predicted teachers’ psychological well-being. Teachers who experience high job demands, low job control and low social support were more likely to report high levels of depression, anxiety and stress. This is in line with previous findings by Doest, Maes, Gebhardt, Koelewijn [87], Bakker & Demerouti [88] as well as with JDC and JDCS models [40,89]. Our findings also showed that social support only moderated the relationship between job demands and anxiety and the relationship between job control and depression. This situation indicates that teachers experience high anxiety when they have high job demands and low social support. Social support plays a significant role as a moderator of psychological distress when there is a match between stress and support received by employees [90,91]. Consistent with previous studies, social support can reduce the negative psychological and physical effects of high work demands and low job control [92]. Social support can help individuals to cope with their job demands and can prevent the effects of psychological stress [90,93,94]

In addition, teachers who receive high social support are less affected by negative emotional effects and can create a positive work environment [76] as well as help teachers effectively in tasks and learning [95]. In line with the JDCS model, social support is predicted to act as a buffering or moderator variable to mitigate the adverse effects of high work strain [89,96]. Isolation (“Iso-strain” jobs) is considered dangerous to mental health because it exhibits the job characteristics that are high job demands, low job control, and low social support [96]. DeClercq [97] argues that social support can reduce the high stress (high job demand and low job control) and prevent or buffer the potential hazards of the job. Furthermore, the “buffer” hypothesis [72] from the JDCS model states that social support moderates the adverse effects of a high-strain work environment.

## 6. Conclusions

In conclusion, this study has successfully identified the significant predictors of teachers’ psychological well-being and the role of job control and social support as a moderating variable to teachers’ psychological well-being in Malaysia. Descriptively, the results showed that the level of job control, job demands and social support categorized as medium. Job demands, job control and social support significantly predict depression. Using the inferential statistics using HLRM, in the first step of the analysis, this study found that the control variables did not contribute significantly to anxiety. Adding job demands, job control, and social support in step two contributed 11.1 percent of the variance in explaining anxiety. This study showed that social support moderated the relationship between job demands and anxiety in the third step. It means that the interaction led to a significant improvement in this regression model. Meanwhile, the two-way interaction model for job control and social support was not significant for anxiety. This study indicated that the control did not contribute significantly to the stress. In addition, the results of the analysis showed job demands, job control, and social support contributed significantly to teachers’ stress. However, social support did not moderate the relationship between job demands and stress as well as job control and stress relationships.

### 6.1. Research Limitation and Future Directions

Despite the study’s contributions, limitations in terms of generalizing its findings need to be considered. Future studies should involve various occupations rather than investigating a specific occupation. In addition, reliance on self-reported measures is another concern of bias in which the generalizing perceptions of demands, control, and support at the individual level. The design of the study was cross-sectional, which prevents the possibility of drawing causal inferences like longitudinal research designs and involves Malaysian respondents with a collectivistic eastern culture. In addition, it would be more valuable when future studies could explain how respondents’ (Muslim) “culture” as a form of social support in the study coped (or not) with job demands. Furthermore, how an Eastern model (compared with a Western model) of analysis could be more helpful in supporting these teachers’ psychological wellbeing.

### 6.2. Research Implications

The present study highlighted the importance of the psychosocial work environment that enhances teachers’ psychological well-being. Organizations, especially administrators, should cooperate in providing positive work environments characterized by low job demands, high job control and high social support. Although numerous studies have focused on psychological well-being based on JCD and JCDS models, few studies have evaluated the Western model in a multi-ethnic collectivistic culture like Malaysia. Moreover, extending the generalization of the Western model may enrich the corpus of literature in the field. The result of this study provides insights and contributes to the literature of teachers’ psychological well-being determinants in the context of Malaysia.

## Figures and Tables

**Table 1:** Respondent’s distribution.

Category	Items	Frequency	Percentage
Gender	Male	78	23.3
	Female	257	76.7
Age	21–30 years	15	4.5
	31–40 years	102	30.44
	41–50 years	154	45.96
	51–60 years	64	19.1
Ethnicity	Malay	323	96.4
	Chinese	9	2.7
	Indian	3	0.9
Marital status	Single	20	6
	Married	307	91.6
	Divorcee	3	0.9
	Widower	5	1.5
No. of children	Nil	35	10.4
	1	28	8.4
	3	57	17
	4	70	20.9
	5 or more	109	32.5
Working experience	3–5 years	10	3
	6–8 years	23	6.9
	9–10 years	34	10.1
	11 years or more	268	80

**Table 2:** Scoring of psychological well-being (DASS).

Severity Categories	Scoring		
	Depression	Anxiety	Stress
Normal	0–4	0–3	0–7
Mild	5–6	4–5	8–9
Moderate	7–10	6–7	10–12
Severe	11–13	8–9	13–16
Extremely severe	14+	10+	17+

**Table 3:** The results of descriptive statistics and level of psychological well-being.

	Variable(s)	Mean	Std Deviation	Level
Psychological well-being	Depression	28.06	2.47	Extremely Severe
	Anxiety	27.47	2.72	Extremely Severe
	Stress	23.39	2.40	Extremely Severe

**Table 4:** The results of the severity level of psychological well-being (depression, anxiety and stress).

Severity Categories	Psychological Well-Being		
	Depression	Anxiety	Stress
Normal	0 (0.00)	0 (0.00)	22 (6.57)
Mild	2 (0.57)	3 (0.89)	46 (6.57)
Moderate	101(30.15)	6 (1.79)	132 (13.73)
Severe	146 (43.59)	50 (14.93)	127 (39.40)
Extremely severe	86 (25.69)	276 (82.39)	8 (2.39)

Note: double brackets () indicates percentage.

**Table 5:** The results of the descriptive statistics and level of job control, job demands and social support.

Variable(s)	Mean	Std Deviation	Level
Job control	3.26	0.30	Medium
Job demands	2.72	0.32	Medium
Social support	3.07	0.22	Medium

**Table 6:** The results of correlation between all studied variables.

No.	Variable(s)	Cronbach Alpha	1	2	3	4	5	6
1	Depression	0.86	1.000					
2	Anxiety	0.88	0.70 **	1.000				
3	Stress	0.86	0.78 **	0.73 **	1.000			
4	Job demands	0.65	0.22 **	0.20 **	0.21 **	1.000		
5	Job control	0.79	−0.21 **	−0.23 **	−0.24 **	0.22 **	1.000	
6	Social support	0.84	−0.07 *	−0.07 *	−0.07 *	−0.01	0.20 **	1.000

Note: *n* = 335., sig. at the level * *p* < 0.05; ** *p* < 0.01; *** *p* < 0.001.

**Table 7:** The results of hierarchical linear regression model in predicting depression.

Variable(s)	Standardized Coefficient (β)		
	1	2	3
Control variables			
Gender	0.082	0.071	0.075
Age	−0.042	−0.039	−0.889
Marital status	−0.028	−0.010	−0.075
No. of children	−0.002	−0.014	−0.010
Work experience	0.082	0.086	0.077
Predictor variables			
Job demands		0.171 **	0.163 **
Job control		−0.197 ***	−0.209 ***
Social support		−0.110 *	−0.132 *
Two-way interaction			
Job demands × social support			0.064
Job control × social support			0.117 *
R^2^	0.019	0.104	0.122
∆R^2^	0.019	0.085	0.018
F Change	1.241	10.335 ***	3.398 *

Note: *n* = 335., sig. at the level * *p* < 0.05; ** *p* < 0.01; *** *p* < 0.001.

**Table 8:** The results of hierarchical linear regression model in predicting anxiety.

Variable(s)	Standardized Coefficient (β)		
	1	2	3
Control variables			
Gender	0.103	0.093	0.095
Age	−0.015	−0.012	−0.019
Marital status	−0.031	−0.012	−0.003
No. of children	−0.024	−0.034	−0.032
Work experience	−0.088	−0.095	−0.101
Predictor variables			
Job demands		0.143 ***	0.134 ***
Job control		−0.222 **	−0.228 **
Social support		−0.109 *	−0.113 *
Two-way interaction			
Job demands × social support			0.110 *
Job control × social support			0.061
R^2^	0.026	0.111	0.128
∆R^2^	0.026	0.085	0.017
F Change	1.760	10.414 ***	3.104 *

Note: *n* = 335., sig. at the level * *p* < 0.05; ** *p* < 0.01; *** *p* < 0.001.

**Table 9:** The results of hierarchical linear regression model in predicting stress.

Variable(s)	Standardized Coefficient (β)		
	1	2	3
Control variables			
Gender	0.051	0.040	0.044
Age	−0.051	−0.048	−0.056
Marital status	−0.025	−0.005	−0.002
No. of children	0.006	−0.005	−0.002
Work experience	−0.046	−0.054	−0.044
Predictor variables			
Job demands		0.157 **	0.152 **
Job control		−0.234 ***	−0.244 ***
Social support		−0.122 *	−0.141 *
Two-way interaction			
Job demands x social support			0.031
Job control x social support			0.092
R^2^	0.011	0.109	0.118
∆R^2^	0.011	0.098	0.009
F Change	0.707	11.995 ***	1.713

Note: *n* = 335., sig. at the level * *p* < 0.05; ** *p* < 0.01; *** *p* < 0.001.

## Data Availability

Not applicable.

## Appendix A. Work Environment and People You Work with

**Item(s)**
1. My job requires that I learn new things.
2. My job involves a lot of repetitive work.
3. My job requires me to be creative.
4. My job requires a high level of skill.
5. I get to do a variety of different things on my job.
6. I have an opportunity to develop my own special abilities.
7. My job allows me to make a lot of decisions on my own.
8. In my job, I have very little freedom to decide how I do my work.
9. I have a lot of say about what happens in my job.
10. My job requires working very fast.
11. My job requires working very hard.
12. I am not asked to do an excessive amount of work.
13. I have enough time to get the job done
14. I am free from conflicting demands that others make.
15. My supervisor is concerned about the welfare of those under him.
16. My boss pays attention to what I am saying.
17. My boss is helpful in getting the job done.
18. My boss is successful in getting people to work together.
19. People I work with are competent in doing their jobs.
20. People I work with take a personal interest in me.
21. People I work with are friendly.
22. People I work with are helpful in getting the job done.

## Appendix B. Psychological Well-Being (Depression, Anxiety and Stress)

**Item(s)**
1. I found it hard to wind down
2. I was aware of dryness of my mouth
3. I couldn’t seem to experience any positive feeling at all
4. I experienced breathing difficulty (e.g., excessively rapid breathing, breathlessness in the absence of physical exertion)
5. I found it difficult to work up the initiative to do things
6. I tended to over-react to situations
7. I experienced trembling (e.g., in the hands)
8. I felt that I was using a lot of nervous energy
9. I was worried about situations in which I might panic and make a fool of myself
10. I felt that I had nothing to look forward to
11. I found myself getting agitated
12. I found it difficult to relax
13. I felt down-hearted and blue
14. I was intolerant of anything that kept me from getting on with what I was doing
15. I felt I was close to panic
16. I was unable to become enthusiastic about anything
17. I felt I wasn’t worth much as a person
18. I felt that I was rather touchy
19. I was aware of the action of my heart in the absence of physical exertion (e.g., a sense of heart rate increase, heart missing a beat)
20. I felt scared without any good reason
21. I felt that life was meaningless
